# Bone marrow mesenchymal stem cells combine with normothermic machine perfusion to improve rat donor liver quality—the important role of hepatic microcirculation in donation after circulatory death

**DOI:** 10.1007/s00441-020-03202-z

**Published:** 2020-04-29

**Authors:** Liu Yang, Huan Cao, Dong Sun, Bin Hou, Ling Lin, Zhong-Yang Shen, Hong-Li Song

**Affiliations:** 1 Tianjin First Central Hospital Clinic Institute, Tianjin Medical University, Tianjin, 300070 People’s Republic of China; 2 Department of Organ Transplantation, Tianjin First Central Hospital, No. 24 Fukang Road, Nankai District, Tianjin, 300192 People’s Republic of China; 3 NHC Key Laboratory of Critical Care Medicine, Tianjin, People’s Republic of China; 4 Tianjin Clinical Research Center for Organ Transplantation, Tianjin, People’s Republic of China; 5 Key Laboratory of Transplant Medicine, Chinese Academy of Medical Sciences, Tianjin, People’s Republic of China; 6 Tianjin Key Laboratory of Organ Transplantation, Tianjin, People’s Republic of China

**Keywords:** Bone marrow mesenchymal stem cells, Donation after circulatory death, Normothermic machine perfusion, Ischemia–reperfusion injury, Microcirculation

## Abstract

Donation after circulatory death (DCD) can expand the donor pool effectively. A gap remains in outcome between DCD livers and living donor livers, warranting improved DCD liver quality and urgent resolution. Bone marrow mesenchymal stem cells (BMMSCs) can regulate immunity, participate in the anti-inflammatory response, and secrete cytokines. We investigated the effect of BMMSCs combined with normothermic machine perfusion (NMP) on DCD liver quality, and the role of microcirculation therein. Rat thoracic aortas were clipped to obtain DCD livers, and a rat NMP system was established. The DCD livers were grouped by preservation method: normal, static cold storage (SCS), NMP (P), and BMMSCs plus NMP (BP); storage time was up to 8 h. Liver function in outflow perfusate was detected by biochemical methods; liver tissue histopathology was observed by hematoxylin–eosin staining; hepatocyte ultrastructure was observed by transmission electron microscopy; hepatocyte apoptosis was detected by terminal deoxynucleotidyl transferase dUTP nick end labeling; liver microcirculation–related indicators were detected by immunofluorescence, immunohistochemistry, Western blotting, and enzyme-linked immunosorbent assay. Compared with SCS, P and BP significantly improved liver function and liver histological damage, reduced hepatocyte apoptosis, and repaired hepatocyte mitochondrial damage after 6 h in vitro. BP also significantly inhibited intrahepatic macrophage activation and intercellular adhesion, improved endothelial damage, and significantly improved endothelin 1–nitric oxide balance and microcirculation perfusion. In conclusion, BP can improve DCD liver microcirculation and quality. The mechanism may be the improvement of improve hepatic sinusoidal endothelial injury and microcirculation perfusion by inhibiting macrophage activation and intercellular adhesion.

**Electronic supplementary material:**

The online version of this article (10.1007/s00441-020-03202-z) contains supplementary material, which is available to authorized users.

## Introduction

Liver transplantation is the only effective treatment for end-stage liver disease (de Haas et al. 2018). However, in the past decade, the world has faced a serious shortage of donor organs. To expand the donor pool, the use of extended criteria donors has increased (Manyalich et al. 2018), including donation after circulatory death organs (DCD), which has become an effective means of expanding the donor pool and is currently a research hotspot on donor issues (Yeh and Uygun 2019). In particular, DCD livers experience longer periods of warm ischemia and ischemia–reperfusion injury (IRI) compared with donation after brain death (DBD) livers. Therefore, DCD livers have higher risk of early postoperative allograft dysfunction, primary nonfunction, ischemic cholangiopathy, and vascular embolism than DBD livers, greatly affecting donor liver survival and patient prognosis (Angelico et al. 2018; DeOliveira et al. 2011); these problems have not been resolved. Static cold storage (SCS) is subject to some limitations for preserving DCD livers, which may not have sufficient physiological reserves for tolerating SCS-related damage; liver energy metabolism and mitochondrial function are easily impaired, and are particularly sensitive to SCS-related IRI; accordingly, SCS exposure should be restricted (Detelich and Markmann 2018a; Ferrigno et al. 2017). Normothermic machine perfusion (NMP) can simulate the normal metabolic state in vivo, can reveal the quality of the liver by assessing bile production and lactate clearance, and store and repair donor livers. NMP also has strong advantages for storing high-risk and marginal donor livers, and increases the use of donor livers effectively, which is promising for donor pool expansion (Laing et al. 2017b; Watson et al. 2017).

Liver biosynthesis, metabolism, transformation, and defense are all dependent on hepatic microcirculation. Changes in the liver microcirculatory structure and function greatly affect liver physiological function. Microcirculatory disorders are the determinants of liver damage; acute liver injury, IRI, and inflammation can cause liver microcirculatory disorders (Vollmar and Menger 2009; Gracia-Sancho et al. 2019). Bone marrow mesenchymal stem cells (BMMSCs) are a class of non-hematopoietic stem cells derived from stromal cells that can regulate immunity, participate in the anti-inflammatory response, and secrete cytokines (Yang et al. 2016a, b; Zheng et al. 2018; Marquez-Curtis et al. 2015; Sassoli et al. 2018), which can inhibit the macrophage-mediated inflammatory response (Li et al. 2016) and improve IRI (Chu et al. 2019). BMMSC transplantation can reduce hepatic IRI and inhibit hepatocyte apoptosis in transplanted livers (Wu et al. 2016). Here, we explored the effect of combining BMMSCs with NMP on DCD liver quality, and studied the changes in donor liver microcirculation, which could provide an experimental basis for improving the quality of DCD livers.

## Materials and methods

### Animals and materials

We purchased specific pathogen-free (SPF) rats from the China Food and Drug Administration (Beijing, China). The animals were kept for 2 weeks at 50% humidity, 18–23 °C, and under 12 h light–dark, with ad libitum access to food and water. We replaced the cages and bedding regularly. All animals received humane care in compliance with the National Institutes of Health Guide for the Care and Use of Laboratory Animals (8th edition); the Animal Care and Research Committee of Tianjin First Central Hospital (Tianjin, China) approved all protocols (Permit number: 2016-03-A1). BMMSCs were extracted from healthy male Sprague-Dawley (SD) rats (*n* = 15, 4–5 weeks old, 40–60 g); DCD livers were obtained from healthy male SD rats (*n* = 50, 6–8 weeks old, 200–220 g).

### Reagents and instruments

Dulbecco’s modified Eagle’s medium (DMEM)/F12 medium (1:1); 0.25% trypsin–EDTA solution (Gibco, Carlsbad, CA, USA); penicillin–streptomycin solution (HyClone, Logan, UT, USA); fetal bovine serum (FBS; Biowest, Loire Valley, France); BMMSC surface marker-related antibodies [anti-rat CD34–fluorescein isothiocyanate (FITC), anti-rat CD29–phycoerythrin (PE), anti-rat CD45–PE, anti-rat CD90–FITC, anti-rat RT1A–PE, anti-rat RT1B–FITC (BioLegend, San Diego, CA, USA)]; adipogenic and osteogenic differentiation medium (Sigma Aldrich, Merck KGaA, St. Louis, MO, USA); Oil Red O (Beijing Dingguo Changsheng Biotechnology, Beijing, China), von Kossa cell staining kit (Genmed, Shanghai, China); rat green fluorescent protein genomic adenovirus (GFP-Adv, GeneChem, Shanghai, China); radioimmunoprecipitation assay (RIPA) lysis buffer (Beijing Solarbio Science & Technology, Beijing, China); bicinchoninic acid (BCA) protein assay kit, sodium dodecyl sulfate–polyacrylamide gel electrophoresis (SDS-PAGE) kit (Beyotime, Shanghai, China); streptavidin–peroxidase kit (ZSGB-BIO, Beijing, China); polyvinylidene fluoride (PVDF) membrane, electrochemiluminescence solution (Millipore, Billerica, MA, USA); In Situ Cell Death Detection Kit (Roche, Basel, Switzerland); von Willebrand factor (vWF) mouse antibody, vascular cell adhesion molecule-1 (VCAM-1) mouse antibody (Santa Cruz Biotechnology, Santa Cruz, CA, USA); endothelial nitric oxide synthetase (eNOS) rabbit antibody (Cell Signaling Technology, Boston, MA, USA); CD14 rabbit antibody, inducible nitric oxide synthetase (iNOS) rabbit antibody, intercellular cell adhesion molecule-1 (ICAM-1) rabbit antibody (Proteintech, Wuhan, China); CD68 rabbit antibody, endothelin-1 (ET-1) rabbit antibody (BIOSS, Beijing, China); glyceraldehyde-3-phosphate dehydrogenase (GAPDH) rabbit antibody (SAB, College Park, MD, USA); goat anti-rabbit immunoglobulin G–horseradish peroxidase (IgG–HRP), goat anti-mouse IgG–HRP, FITC-conjugated goat anti-rabbit IgG or Alexa Fluor 488-conjugated goat anti-rabbit IgG (ZSGB-BIO); ET-1, NO, VCAM-1, thrombomodulin (TM), platelet-activating factor (PAF) enzyme-linked immunoassay (ELISA) kit (Tianjin Anoric Biotechnology, Tianjin, China); ICAM-1 ELISA kit (Lianke Biotech, Hangzhou, China); inverted fluorescent microscope (Olympus, Tokyo, Japan); Eclipse Ni-U positive fluorescence microscope (Nikon, Tokyo, Japan); BD Accuri C6 Plus flow cytometer (BD Biosciences, Franklin Lakes, NJ, USA); Molecular Imager ChemiDoc XRS+ system (Bio-Rad, Foster City, CA, USA).

### BMMSC isolation, culture, and identification

The rats were sacrificed by cervical dislocation after sevoflurane anesthesia, in accordance with the Canadian Council on Animal Care (CCAC) guidelines on euthanasia of animals used in science; the femur and tibia were removed aseptically. The marrow cavity was rinsed with DMEM/F12 (1:1) containing 10% FBS; the cell suspension was inoculated into T75 culture flasks, and cultured at 37 °C with 5% CO_2_. Well-grown passage 3 cells were resuspended for detection and backup, labeled with fluorescent antibodies: anti-CD29-PE, anti-CD34-FITC, anti-CD45-PE, anti-CD90-FITC, anti-RT1A-PE, and anti-RT1B-FITC, and incubated for 30 min in the dark for flow cytometry.

Well-grown passage 3 BMMSCs were cultured continuously in adipogenic differentiation medium, which was changed every 3 days. After 8–10 days, the BMMSCs underwent 30-min Oil Red O staining, and were rinsed with phosphate-buffered saline (PBS) before being observed under microscopy.

Well-grown passage 3 BMMSCs were also cultured continuously in osteogenic differentiation medium, which was changed every 3 days. After 13–15 days, the BMMSCs underwent von Kossa staining and were observed under microscopy.

### BMMSC colonization in the liver

For acquiring GFP-BMMSCs, the spent culture medium was removed from well-grown passage 3 BMMSCs and replaced with 5 mL DMEM/F12 per flask. Subsequently, GFP-Adv transfection solution was added at a multiplicity of infection (MOI) of 10. After 6 h, complete medium was added, and the culture medium was changed every other day. After 72 h, the proportion of GFP-expressing cells was observed under fluorescence microscopy. BMMSC colonization was detected in frozen sections from GFP-BMMSC plus 6-h NMP (protected from light during perfusion) livers: at the end of the 6-h NMP, about 1 × 1 × 0.5 cm^3^ liver tissue blocks were randomly removed and embedded in glue, quick-frozen in liquid nitrogen. Sections (10-μm-thick) were obtained, fixed in 4% paraformaldehyde, and observed under fluorescence microscopy.

### Rat DCD liver acquisition and establishment of liver NMP system

The rats were fasted for 12 h, but allowed access to water. The rats were anesthetized by intraperitoneal injection with 2% pentobarbital sodium (0.3 mL/100 g body weight). The liver was exposed using an abdominal median incision. The perihepatic ligaments were dissected, then the left subphrenic vein, right renal vein, adrenal venous plexus, and hepatic artery were ligated. Then, the portal vein was separated, the pyloric vein and splenic vein ligated, and the bile duct was inserted. After the diaphragm was opened, the thoracic aorta was clipped, and the heart was pressed with a cotton swab to simulate cardiac death. The abdominal cavity was covered with gauze soaked in warm saline for 30 min, then the liver was harvested and weighed for wet weight.

The NMP system is a single-cycle system that mainly includes centrifugal pumps, membrane oxygenators, organ chambers, heaters, and pressure and temperature monitors. The DCD liver was placed in the organ chamber and the perfusion system was connected in advance. Perfusate was oxygenated through a membrane oxygenator and flowed through the portal vein, continuously perfused at a rate of 2 mL/g/min (by liver wet weight). Portal pressure was maintained at 10–12 mm H_2_O and monitored by pressure sensor. The NMP system was maintained at 35–38 °C. The perfusate ingredients were 60 mL DMEM/F12 (1:1) containing 20% FBS and 1% penicillin–streptomycin solution (penicillin 10,000 U/mL, streptomycin 10,000 μg/mL), 20 mL fresh blood, 5 U/mL heparin, 2 U/L insulin, and 2.5 μg/mL dexamethasone (Supplementary Fig. 1).

### Groups and treatments

The DCD livers were grouped based on the preservation method: Normal, SCS, NMP alone (P), and BMMSCs plus NMP (BP). In the normal group, serum and livers were obtained to use. The blood was washed from the SCS livers with 20 mL 4 °C University of Wisconsin solution (UW) and stored at 4 °C in UW; the livers were harvested after 4 h, 6 h, and 8 h. In the NMP group, 2 mL normal saline was injected via the portal vein immediately after the NMP system was connected, and NMP was performed continuously. In the BP group, 2 mL medium suspension containing 1 × 10^7^ BMMSCs was injected via the portal vein immediately after the NMP system was connected. Five DCD livers per group were used at each time point. Inflow perfusate was collected for blood gas analysis at the instant of perfusion, 2 h, 4 h, 6 h, and 8 h; outflow perfusate centrifuged to obtain the supernatant. Liver specimens were collected at the instant of perfusion, 2 h, 4 h, 6 h, and 8 h; liver tissues were randomly fixed in formalin or 2.5% glutaraldehyde solution; other liver tissues were minced and quick-frozen with liquid nitrogen. All samples were stored at − 80 °C for testing.

### Liver function

We measured alanine aminotransferase (ALT), alkaline phosphatase (ALP), albumin (ALB), aspartate aminotransferase (AST), and mitochondrial AST (ASTm) levels with an automated biochemical analyzer (Hitachi, Tokyo, Japan) using the instructions of the manufacturer.

### Liver histopathological, immunohistochemical, and immunofluorescence staining

For hematoxylin–eosin (HE) staining, tissue slides were baked for 1 h at 70 °C and underwent deparaffinization with dimethylbenzene, gradient ethanol hydration, HE staining, gradient ethanol dehydration, and were mounted with neutral balsam. The hepatic pathological changes were observed under light microscopy. Hepatic IRI severity was evaluated based on Suzuki’s criteria and graded according to a scale of 0–4 (Suzuki et al. 1993) (Table 1).

**Table 1:** Suzuki’s histological criteria

Numerical assessment	Congestion	Vacuolization	Necrosis
0	None	None	None
1	Minimal	Minimal	Single-cell necrosis
2	Mild	Mild	< 30%
3	Moderate	Moderate	30–60%
4	Severe	Severe	> 60%

For immunohistochemical (IHC), tissue slides were processed as above up to gradient ethanol hydration, and underwent antigen retrieval and blocking with normal goat serum. The slides were incubated with primary antibodies (1:50), biotinylated goat anti-mouse/rabbit IgG polymer, and streptavidin working solution labeled with HRP. Then, they were stained with diaminobenzidine and hematoxylin, underwent gradient alcohol dehydration, and were mounted with neutral balsam. The ET-1, eNOS, iNOS, ICAM-1, VCAM-1, and vWF levels were observed.

For immunofluorescence, tissue slides were processed as above up to blocking with normal goat serum. The slides were incubated with primary antibodies (1:50) and the secondary antibodies FITC-conjugated goat anti-rabbit IgG or Alexa Fluor 488–conjugated goat anti-rabbit IgG. The CD14 and CD68 levels were observed.

### In situ cell death detection (terminal deoxynucleotidyl transferase dUTP nick end labeling [TUNEL])

Liver tissue slides were baked at 70 °C for 1 h, deparaffinized with dimethylbenzene, hydrated in gradient ethanol, and permeabilized by 10 μg/mL proteinase K (Beijing Solarbio Science & Technology) at 37 °C for 20 min. Then, the slides were washed with PBS, and reaction solution (labeling solution:enzyme solution = 50:1) was added and incubated at 37 °C for 1 h. The nuclei were stained with 4′6-diamidino-2-phenylindole (DAPI), and hepatocyte apoptosis was observed.

### Electron microscopy

Fresh liver tissue was cut into 1 × 1 × 2 mm^3^ samples, fixed in 2.5% glutaraldehyde solution, embedded, and sliced into ultrathin sections. The ultrastructural changes were observed under a H-600 transmission electron microscope (Hitachi).

### Western blotting

Total liver protein was extracted from RIPA lysis buffer; the total protein concentration was detected by the BCA method. The proteins were separated electrophoretically and wet-transferred to PVDF membranes, blocked for 1 h with 5% skimmed milk (BD Biosciences), then incubated with primary antibody against CD14 (1:500), CD68 (1:500), ET-1 (1:500), eNOS (1:1000), iNOS (1:500), ICAM-1 (1:500), VCAM-1 (1:100), vWF (1:500), and GAPDH (1:3000) at 4 °C overnight. The membranes were rinsed with TBST buffer (solution containing tris, NaCl, and Tween 20), and incubated with secondary antibody (1:2000) at room temperature for 1 h. The membranes were exposed using the Bio-Rad Molecular Imager ChemiDoc XRS+ system; image grayscale values were analyzed using AlphaView SA 3.4.0.0 (ProteinSimple, San Jose, CA, USA) to calculate the relative protein expression.

### ELISA

ET-1, NO, VCAM-1, ICAM-1, TM, and PAF levels in the outflow perfusate were tested according to the kit manufacturer’s protocol.

### Statistical analysis

The data were analyzed using SPSS 17.0 (SPSS, Chicago, IL, USA). Means ± standard deviation were used to present normally distributed data. One-way analysis of variance was used to assess the significance of differences between groups; least significant difference and Student–Newman–Keuls post hoc comparison were used for further comparisons. Statistically significant differences were indicated using *p* < 0.05. Data were plotted for presentation using GraphPad Prism 5.0 (GraphPad, La Jolla, CA, USA).

## Results

### BMMSC morphology and identification

**Fig. 1:**
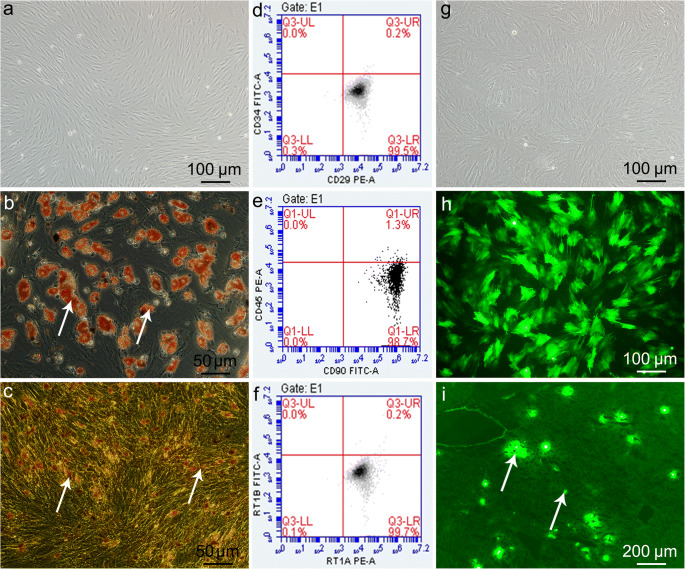
BMMSC morphology, phenotype, differentiation characteristics, and colonization. **a** Passage 3 BMMSCs (scale bar = 100 μm). **b** BMMSC adipogenic differentiation shows typical red lipid droplets in cells (white arrows; scale bar = 50 μm). **c** BMMSC osteogenic differentiation shows intracellular black calcium salt deposition (white arrows; scale bar = 50 μm). **d**–**f** Flow cytometry of BMMSC surface markers: CD29, CD34, CD45, CD90, RT1A, RT1B. **g** GFP-transfected BMMSCs, i.e., GFP-BMMSCs, in bright field (scale bar = 100 μm). **h** GFP-BMMSCs in fluorescence field; GFP-expressing BMMSCs was > 85% (scale bar = 100 μm). **i** GFP-BMMSCs (white arrows) plus 6-h NMP DCD liver frozen section in fluorescence field; BMMSCs colonized the hepatic sinusoids (scale bar = 200 μm). *GFP* green fluorescent protein, *BMMSCs* bone marrow mesenchymal stem cells, *DCD* donation after circulatory death

The BMMSCs were long and spindle-shaped, and appeared partially vortexed or chrysanthemum-like, with typical MSC morphological characteristics. Flow cytometry showed that the ratio of CD29^+^CD34^−^ cells, CD90^+^CD45^−^ cells, and RT1A^+^RT1B^−^ cells was 99.5%, 98.7%, and 99.7%, respectively, indicating that the passage 3 BMMSCs were of high purity. Oil Red O staining showed several red lipid droplets in the cytoplasm after adipogenic induction, which was consistent with adipocyte characteristics. Von Kossa staining showed black granular or lumpy calcium deposits in the cytoplasm after osteogenic induction, an osteoblast characteristic. The results indicate that the extracted BMMSCs could differentiate into adipocytes and osteoblasts (Fig. 1a–f).

### BMMSC colonization in hepatic sinusoids under NMP

After GFP-Adv transfection of BMMSCs, there were > 85% GFP-expressing BMMSCs, which proved the successful transfer of the GFP gene into the BMMSCs, and the successful construction of GFP-BMMSCs. In the BP process, BMMSCs colonized the DCD liver continuously; after 6-h perfusion, GFP-BMMSCs (green fluorescence) colonizing the hepatic sinusoids were detected in frozen DCD liver sections. The results suggest that during DCD liver repair, BMMSCs can colonize the hepatic sinusoids to play a corresponding role (Fig. 1g–i).

### Evaluation of rat NMP system

**Fig. 2:**
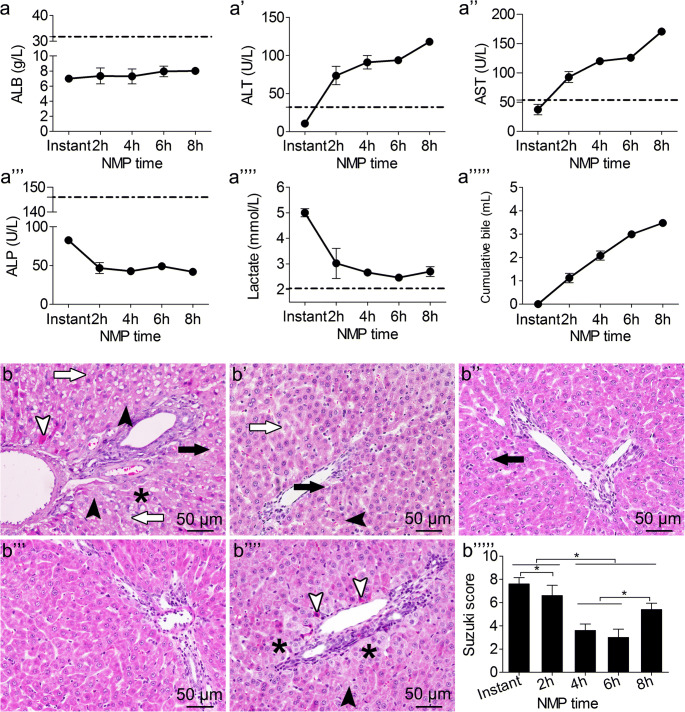
Effects of rat NMP system on DCD liver. (a-a””’) ALB, ALT, AST, ALP levels; lactate clearance; and bile production. ALT and AST levels were significantly different among the time points except for hour 4 and 6. Lactate gradually decreased and showed an increasing trend after hour 6; lactate at hour 6 was significantly lower than that at hour 2. (b–b””) Liver pathology and Suzuki’s scores (b””’): HE-stained liver at: (b) the instant of perfusion, (b’) hour 2 after perfusion, (b”) hour 4 after perfusion, (b”’) hour 6 after perfusion, (b””) hour 8 after perfusion. Liver pathology was best at hour 4 and 6; hepatocyte edema (black asterisks), eosinophilic degeneration (white arrowheads), and hepatocyte necrosis (black arrowheads) were observed at hour 8 (scale bar = 50 μm, *n* = 5) (black arrows indicate hepatocyte vacuolization, white arrows indicate sinusoid congestion). *p < 0.05. *ALB* albumin, *ALT* alanine aminotransferase, *AST* aspartate aminotransferase, *ALP* alkaline phosphatase, *NMP* normothermic machine perfusion, *HE* hematoxylin–eosin, *DCD* donation after circulatory death

ALB levels in the outflow perfusate did not fluctuate significantly with perfusion time, and were not significantly different at each time point. ALT and AST levels showed an increasing trend, which slowed gradually, and the elevations decreased significantly from hour 4 to hour 6. The ALT and AST levels were not significantly different from hour 4 to hour 6, but were significantly different between the other time points. After 6-h perfusion, ALT and AST levels were significantly elevated (*p* < 0.05). ALP decreased gradually; there was no significant difference at each time point. Lactate rapidly decreased to low levels after perfusion, and the lactate decline decreased during the 6-h period. After 6-h perfusion, there was an evident increase in lactate; the difference between the levels at hour 2 and 6 was significant (*p* < 0.05). Bile increased gradually, but decreased after 6-h perfusion (Fig. 2(a-a””’)).

Liver histopathology showed severe cytoplasmic vacuolization immediately after perfusion; the severity of hepatic sinusoid congestion, cell edema, and acidophilic degeneration were more evident. The cytoplasmic vacuolization degeneration, cell edema, and hepatic sinusoid congestion gradually decreased along with perfusion time. At hour 6, there was no obvious cell edema, vacuolization degeneration, acidophilic degeneration, or hepatic sinusoid congestion; liver histopathology was the best at hour 6. Hepatocyte edema, acidophilic degeneration, and necrosis were observed at hour 8. Suzuki’s scores were significantly different at each time point, and were lower at hour 4 and 6, but were not significantly different then. Suzuki’s score increased significantly at hour 8, suggesting that IRI improved gradually with perfusion time, but that the liver appeared injured after 6-h perfusion (Fig. 2(b–b””’)).

Liver function, lactate clearance, bile production, and histopathology were evaluated in the rat NMP system, suggesting that this preservation method could significantly improve DCD liver function and histopathology. The DCD liver quality improved gradually with perfusion time, but liver function, lactate clearance, bile production, and histopathology deteriorated after 6-h perfusion, and DCD liver quality decreased, suggesting that the best and longest time for storing DCD liver in the rat NMP system was 6 h and that further perfusion might affect DCD liver quality.

### BMMSCs plus NMP improved DCD liver quality

#### BMMSCs plus NMP improved DCD liver function

**Fig. 3:**
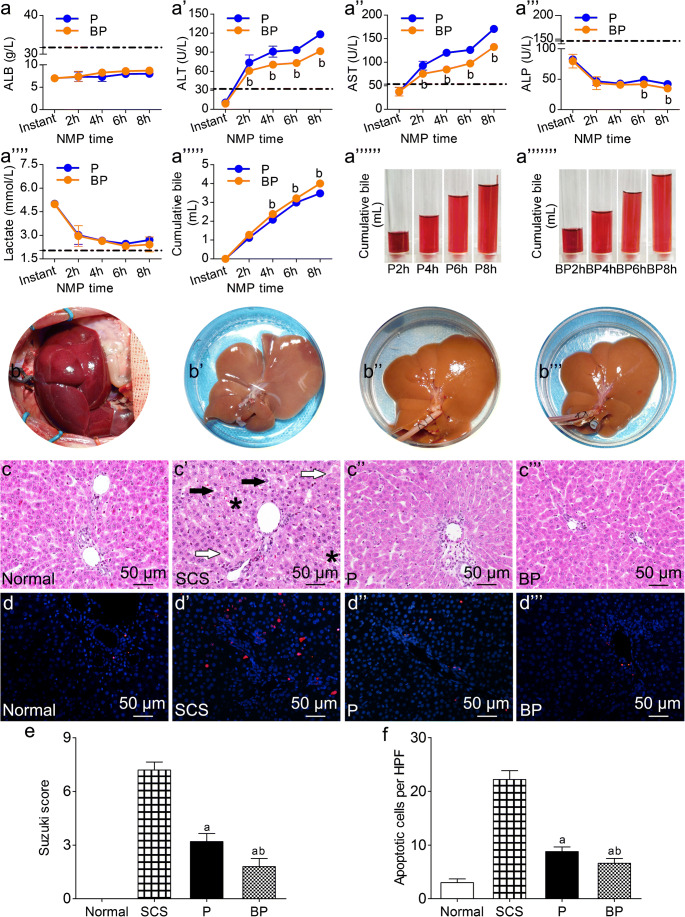
The effects of different preservation methods on DCD liver quality. (a–a”’) Liver function (ALB, ALT, AST, ALP), lactate clearance (a””), and bile levels (a””’–a”””’) (as the perfusate contained phenol red, the excreted bile appeared red, *n* = 5). (b–b”’) DCD liver manifestations: (b) Warm ischemia 30 min, (b’) 6-h SCS, (b”) 6-h NMP, (b”’) 6-h BMMSCs plus NMP. (c-c”’) DCD liver HE staining, (d-d”’)TUNEL staining, Suzuki’s scores (e) and apoptosis statistics (f). The SCS group had severe HE assessment, cell vacuolar degeneration (black arrows), edema (black asterisks), and hepatic sinusoid congestion (white arrows); the BP group and P group had almost no vacuolar degeneration, hepatic sinusoid congestion, or inflammatory cell infiltration (scale bar = 50 μm, *n* = 5). TUNEL: red indicates apoptotic cells; DAPI-labeled nuclei appear blue. (scale bar = 50 μm, *n* = 5). ^a^*p* < 0.05 vs. SCS group, ^b^*p* < 0.05 vs. P group; dashed line indicates the levels in normal rats. *DCD* donation after circulatory death, *SCS* static cold storage, *NMP* normothermic machine perfusion, *BMMSCs* bone marrow mesenchymal stem cells, *HE* hematoxylin–eosin, *TUNEL* terminal deoxynucleotidyl transferase dUTP nick end labeling, *ALT* alanine aminotransferase, *AST* aspartate aminotransferase, *ALP* alkaline phosphatase, *ALB* albumin, *HPF* high-power field, *DAPI* 4′ 6-diamidino-2-phenylindole; P, NMP; BP, BMMSCs plus NMP

The BP group and P group did not have significantly different ALB levels. ALT and AST levels showed an increasing trend, which slowed gradually with perfusion time. The BP group had significantly lower ALT and AST levels than the P group (*p* < 0.05). ALP decreased gradually; the BP group had significantly lower ALP levels than the P group. Liver function testing suggested that BMMSCs plus NMP can improve DCD liver function and quality significantly, and is superior to NMP alone (Fig. 3(a–a”’)).

#### BMMSCs plus NMP promoted lactate clearance and bile production

Lactate levels were highest immediately after perfusion, and decreased gradually to stable levels with perfusion time. After 6-h perfusion, there was an evident increase in lactate; the BP group had lower lactate levels than the P group at each time point. Bile gradually increased with perfusion time. The BP group had significantly higher bile production and rate of increase than the P group at each time point (*p* < 0.05). After 6-h perfusion, the P group had a significantly slower bile increase rate, suggesting that BMMSCs plus NMP can significantly improve DCD liver quality and is superior to NMP alone (Fig. 3(a””–a”””’)).

#### BMMSCs plus NMP improved DCD liver general performance

DCD livers with 30-min warm ischemia were obviously swollen, with severe congestion, uneven texture, purple-red color, and rounded edges. After 6-h SCS, the livers were swollen, with uneven texture, congested, and piebald-like. P and BP livers were not swollen or congested, the texture was uniform, and with soil yellow color, suggesting that NMP alone and combined with BMMSCs can reduce liver swelling and congestion and improve performance (Fig. 3(b–b”’)).

#### BMMSCs plus NMP improved DCD liver histopathology

SCS livers had severe hepatic vacuolar degeneration, edema, and hepatic sinusoid congestion; BP livers had almost no vacuolar degeneration, hepatic sinusoid congestion, or inflammatory cell infiltration, and had less hepatic sinusoid congestion and hepatocyte edema, which was superior to the P livers. Liver pathology improved with perfusion time. The BP group had significantly lower Suzuki’s score than the P group and SCS group (*p* < 0.05). Based on the DCD liver pathologies, BMMSCs plus NMP was the best of the three storage methods, as it could improve liver pathology, and BMMSCs could promote NMP protection of DCD liver (Fig. 3(c–c”’, e)).

#### BMMSCs plus NMP attenuated DCD hepatocyte apoptosis

The normal group had the fewest apoptotic cells, while the SCS group had the most. The P group and BP group had significantly fewer apoptotic cells than the SCS group; the BP group had fewer apoptotic cells than the P group (*p* < 0.05). It is suggested that BMMSCs can attenuate DCD hepatocyte apoptosis, which is superior to NMP alone (Fig. 3(d–d”’, f)).

#### BMMSCs plus NMP alleviated mitochondrial damage in DCD liver

**Fig. 4:**
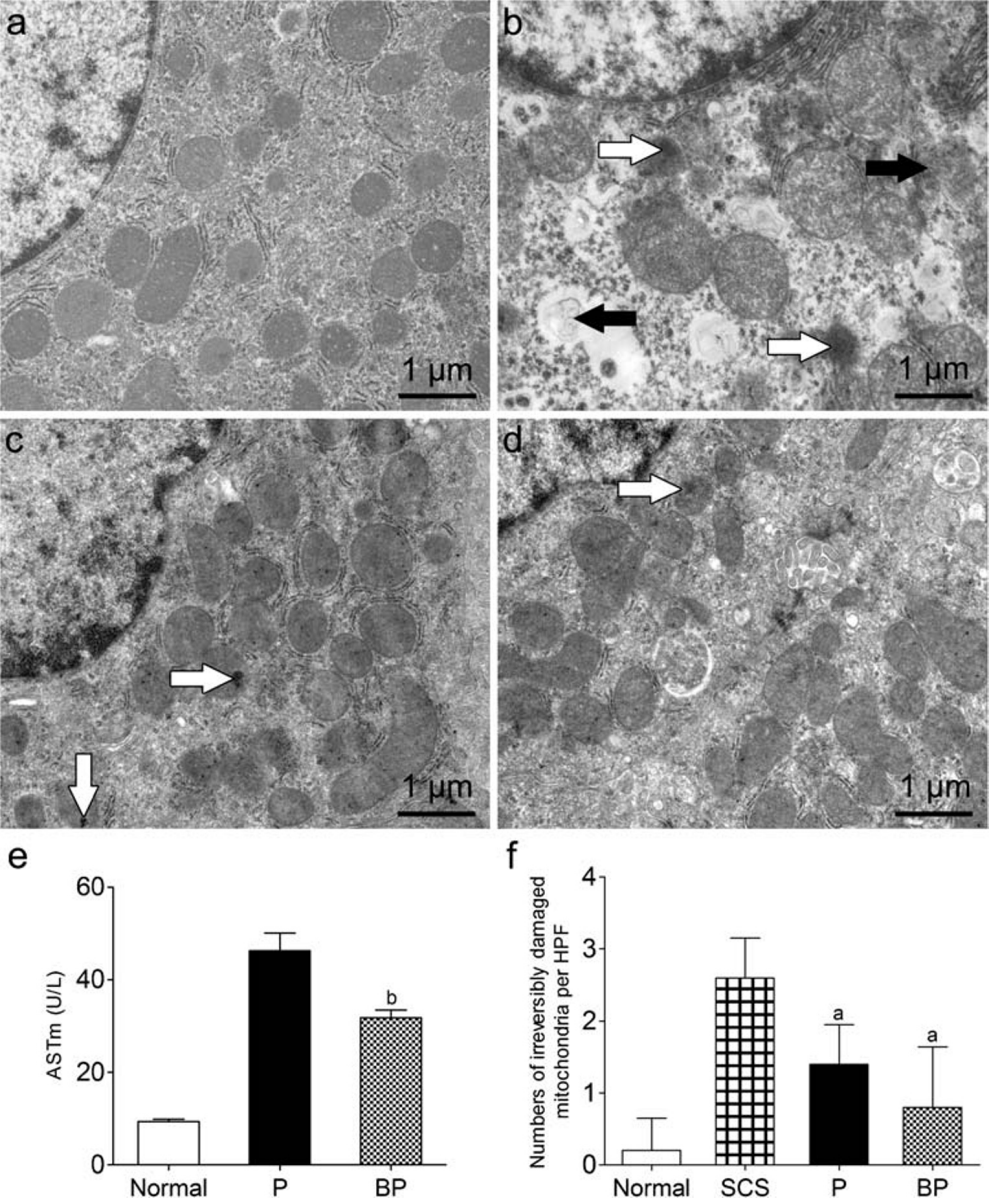
Mitochondrial ultrastructure and damage in DCD liver. **a** Mitochondrial morphology in the (**a**) normal group, **b** SCS group, **c** P group, **d** BP group. The SCS group showed more severe nucleic swelling, mitochondrial edema, vacuolization, irreversible damage (white arrows, flocculated density) and partial mitochondrial lysis (black arrows). Mitochondrial cristae were disrupted (almost disappeared) in SCS liver (scale bar = 1 μm). **e** ASTm levels in perfusate. The PB group had significantly lower ASTm levels than the P group. **f** Number of irreversibly damaged mitochondria per HPF. ^a^*p* < 0.05 vs. SCS group, ^b^*p* < 0.05 vs. P group. *DCD* donation after circulatory death, *SCS* static cold storage, mitochondrial aspartate aminotransferase (ASTm), *HPF* high-power field; P, NMP; BP, BMMSCs plus NMP

Transmission electron microscopy showed more severe nucleic swelling, mitochondrial edema, mitochondrial vacuolization, disrupted mitochondrial cristae (most disappeared), irreversible mitochondrial damage, and partial mitochondrial lysis in the SCS group. The BP group and P group showed no nucleic swelling, mitochondrial swelling, or vacuolization, and had intact mitochondrial cristae and slighter mitochondria damage (Fig. 4a–d). ASTm levels are a marker of mitochondrial damage marker, and were significantly lower in the PB group than in the P group (*p <* 0.05). The SCS group had significantly more irreversibly damaged mitochondria than the P and BP groups (*p* < 0.05) (Fig. 4e). This suggests that BMMSCs plus NMP can improve mitochondrial damage.

### BMMSCs plus NMP improved DCD liver microcirculation

#### BMMSCs plus NMP inhibited macrophage activation

**Fig. 5:**
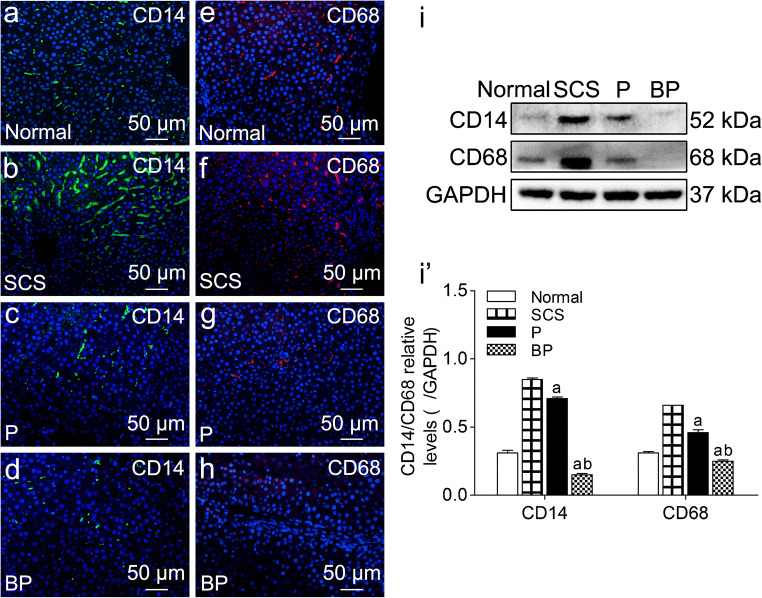
Expression of macrophage surface activating molecules in DCD liver tissues. (a–h) Immunofluorescence staining of CD14 and CD68, green indicates CD14, red indicates CD68, blue indicates DAPI-stained nuclei (scale bar = 50 μm). (i, i’) Western blot and quantitative analysis of CD14 and CD68 (*n* = 5). ^a^*p* < 0.05 vs. SCS group, ^b^*p* < 0.05 vs. P group. *DCD* donation after circulatory death, *SCS* static cold storage; P, NMP; BP, BMMSCs plus NMP

Immunofluorescence staining showed that intrahepatic CD14 and CD68 were expressed in the hepatic sinusoids, and the SCS group had clearly upregulated CD14 and CD68 expression (Fig. 5(a–h)). Quantitative analysis showed that the P group and BP group had significantly lower CD14 and CD68 expression than the SCS group; the BP group had significantly lower expression than the P group (*p* < 0.05), suggesting inhibited intrahepatic macrophage activation in the P group and BP group, and that BMMSCs can inhibit macrophage activation (Fig. 5(i, i’)).

#### BMMSCs plus NMP inhibited cell adhesion and improved endothelial cell damage

**Fig. 6:**
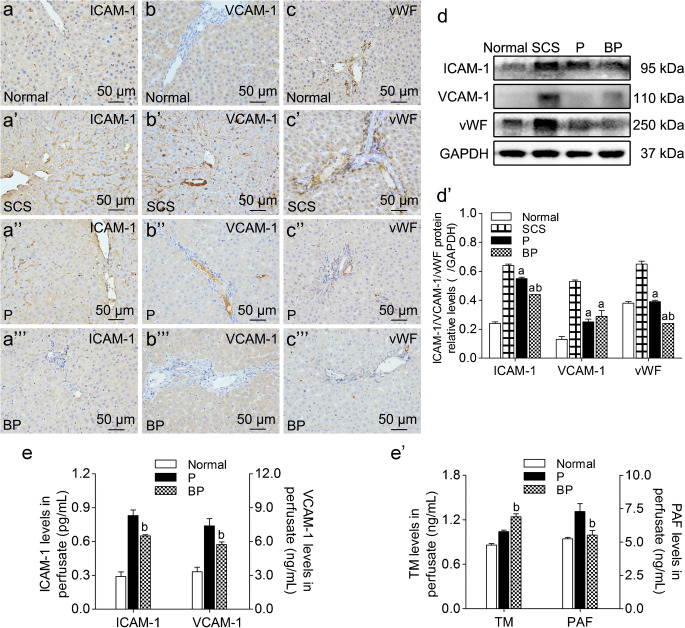
Expression of adhesion molecules and vWF in DCD liver. (a-c”’) IHC of ICAM-1, VCAM-1, and vWF (scale bar = 50 μm). (d, d’) Western blot of vWF, ICAM-1, and VCAM-1 (*n* = 5). (e, e’) Perfusate ICAM-1, VCAM-1, TM, and PAF levels (n = 5). ^a^*p* < 0.05 vs. SCS group, ^b^*p* < 0.05 vs. P group. *ICAM*-*1* intercellular cell adhesion molecule-1, *VCAM*-*1* vascular cell adhesion molecule-1, *vWF* von Willebrand factor, *TM* thrombomodulin, *PAF* platelet-activating factor, *IHC* immunohistochemistry, *DCD* donation after circulatory death, *SCS* static cold storage; P, NMP; BP, BMMSCs plus NMP

ICAM-1 and VCAM-1 are intercellular adhesion molecules. IHC showed that ICAM-1 was mainly expressed in the sinusoidal endothelial cells (SECs), and VCAM-1 was mainly expressed in SECs and vascular endothelial cells. They are the products of the inflammatory reaction and a sign of neutrophil adhesion and infiltration, which also indicates the degree of hepatic sinusoid congestion. The SCS group had significantly higher ICAM-1 and VCAM-1 expression than the P group and BP group; the BP group had significantly lower ICAM-1 expression than the P group (Fig. 6(a–b”’, d, d’)). The BP group had significantly lower perfusate ICAM-1 and VCAM-1 levels than the SCS group and P group (*p* < 0.05). This suggests that BMMSCs can inhibit ICAM-1 and VCAM-1 expression (Fig. 6(e)).

Intrahepatic vWF was mainly expressed in the vascular endothelial cells, and SECs expressed a small amount. The SCS group had an evidently higher proportion of vWF-positive cells than the P group; the proportion was lowest in the BP group. The BP group had significantly lower vWF expression than the SCS group and P group; vWF expression was lower in the P group than in the SCS group (*p* < 0.05). This suggests that BMMSCs can reduce hepatic endothelial damage (Fig. 6(c–c’, d, d’)).

#### BMMSCs plus NMP improved the ET-1/NO balance and microcirculation perfusion

**Fig. 7:**
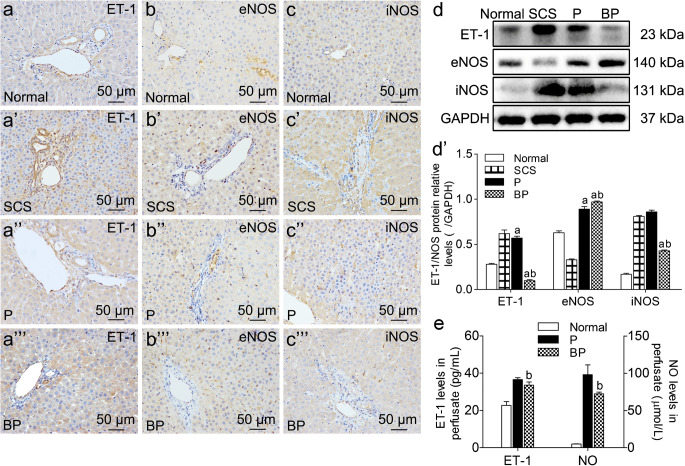
ET-1/NOS expression in DCD liver. (a-c”’) IHC of ET-1/NOS in the Normal, SCS, BP, and P group (scale bar = 50 μm). (d, d’) Western blot and quantitative analysis of intrahepatic ET-1/NOS (*p* < 0.05, n = 5). (e) Perfusate ET-1 and NO levels (*p* < 0.05, *n* = 5). ^a^*p* < 0.05 vs. SCS group, ^b^*p* < 0.05 vs. P group. *ET*-*1* endothelin-1, *eNOS* endothelial nitric oxide synthetase, *iNOS* inducible nitric oxide synthetase, *NO* nitric oxide, *IHC* immunohistochemistry, *DCD* donation after circulatory death, *SCS* static cold storage; P, NMP; BP, BMMSCs plus NMP

IHC showed that ET-1 was expressed in the vessels and hepatic sinusoids. ET-1 expression was lowest in the BP group and was highest in the SCS group; the difference among the three groups was statistically significant (*p* < 0.05). The BP group had significantly lower perfusate ET-1 than the P group (*p* < 0.05). This suggests that BMMSCs inhibit ET-1 expression, reducing hepatic sinusoid contraction and improving hepatic sinusoid perfusion (Fig. 7(a–a”’, d, d’, e)).

Intrahepatic eNOS was expressed in the sinusoidal endothelium and vascular endothelium around the Glisson system. The BP group had significantly higher eNOS expression than the P group and SCS group (*p* < 0.05). iNOS, induced by stress and inflammation, was expressed in the hepatic sinusoids. The BP group had significantly lower iNOS expression than the P group and SCS group; the P group had lower iNOS than the SCS group (*p* < 0.05). This suggests that BMMSCs can promote eNOS synthesis while inhibiting iNOS production in DCD liver (Fig. 7(b–b”’, d, d’)).

NO is mainly synthesized by NOS and is an endothelium relaxing factor that dilates the blood vessels and hepatic sinusoids. Intrahepatic macrophage activation can induce large amounts of iNOS and synthesize large amounts of NO. The BP group had significantly lower perfusate NO levels than the P group (*p* < 0.05). This suggests that BMMSCs can inhibit NO production, indirectly suggesting that BMMSCs can alleviate the stress response and inhibit macrophage activation (Fig. 7(e)).

## Discussion

With the emergence of a shortage of liver donors, DCD donors have increasingly received attention and are growing gradually. However, the presence of warm ischemia injury in DCD livers leads to postoperative complications, rejection, and poor survival outcomes, which presents certain risks and limitations on the use of DCD liver. Therefore, improving the quality of DCD liver is worth studying and requires urgent resolution (Eren et al. 2016). Poor DCD liver quality may be due to (1) insufficient physiological reserve for tolerating SCS-related damage, (2) easily impaired liver energy metabolism and mitochondrial function, and (3) particular sensitivity to SCS-related IRI, so SCS has certain limitations for preserving DCD livers (Detelich and Markmann 2018b; Ferrigno et al. 2017). The emergence of NMP has greatly improved DCD liver quality. NMP preservation is superior to SCS (Ceresa et al. 2017), preventing liver damage caused by cold ischemia effectively, allowing the pre-transplantation evaluation of organ function and significantly improving hepatic IRI, liver vitality, and survival rate, increasing liver utilization effectively (Detelich and Markmann 2018b). However, the total number of NMP applications in clinical liver transplantation is low; most involve studies of animal and abandoned livers; many uncertainties, such as perfusion time, perfusate composition, and oxygen carrier, require further research and optimization (Watson et al. 2017; Burra et al. 2018; Laing et al. 2017a).

Here, we used a stable single-cycle NMP system; perfusion times were at the instant of perfusion, 2 h, 4 h, 6 h, and 8 h. Liver function, lactate clearance, and bile production were detected. ALT and AST increased gradually with perfusion time. During hour 4 and 6 of perfusion, ALT and AST levels declined, but increased significantly after 6 h. Lactate clearance also showed the same trend, which rebounded significantly after 6 h, and the increased bile production was reduced. Hepatic IRI improved gradually after perfusion, and the pathological manifestation was best at hour 4 and 6. Consistent with liver function, hepatocyte edema, hepatic sinusoid congestion, and other pathological manifestations deteriorated after 6 h. To ensure the stability of the research effect, we evaluated the effect of the NMP system on the DCD liver quality comprehensively, and selected the liver from hour 6 with the best quality following NMP preservation.

BMMSCs can alleviate hepatic IRI, reduce hepatocyte damage, accelerate liver regeneration, participate in the anti-inflammatory response, and regulate immunity (Wu et al. 2016; Yang et al. 2016a). We have shown that, when combined with NMP, BMMSCs can colonize DCD liver and have an effect, so we used BMMSCs plus NMP to observe the influence on DCD liver quality and investigate its mechanism. We evaluated the influences of NMP-alone preservation and that of BMMSCs plus NMP on DCD liver quality, and found that for perfusion of up to 6 h, BMMSCs plus NMP was significantly better than NMP alone for improving liver function, promoting lactate clearance, and bile production, significantly improving liver pathology and IRI, and reducing hepatocyte apoptosis. The liver tissue ultrastructural changes showed obvious hepatocyte nuclear edema in the SCS group, and severe mitochondrial swelling and irreversible damage; the BP group had no hepatocyte nuclei or mitochondrial swelling, and had the lowest degree of mitochondrial damage, which verifies the protective role of BMMSCs (Wu et al. 2016; Wang et al. 2017). In hepatic IRI, especially warm ischemia, hepatocyte mitochondria change the earliest. Mitochondria are very sensitive to hypoxia and oxidative stress, and are susceptible to damage. The parameter most closely related to hepatocyte mitochondrial function is hepatic microcirculation stability, for avoiding hypoxia and reactive oxygen species (ROS) damage (Peralta et al. 2013; Melser et al. 2015), Therefore, we researched hepatic microcirculation further.

Liver microcirculation disorder is the determinant of liver damage, and its occurrence will aggravate existing liver disease states such as liver failure, IRI, inflammatory response, small liver syndrome, and portal hypertension, and significantly increase the incidence and mortality of these disease states (Vollmar and Menger 2009). The mechanisms of hepatic microcirculation disorder include microcirculatory hyperinflammatory response caused by macrophage activation, microvascular leukocyte aggregation, disordered ET-1/NO balance, arterial spasm and hepatic sinusoid congestion, and microcirculation occlusion (Vollmar and Menger 2009; Bhogal et al. 2011). We examined the macrophage activation markers CD14, CD68, iNOS, and NO (Cai et al. 2013; van den Berg et al. 2001; Dixon et al. 2013; Cutrn et al. 2002) and found that BMMSCs significantly downregulated their expression and inhibited macrophage activation significantly. BMMSC inhibition of DCD liver macrophage activation under NMP was the key to improving liver microcirculatory disorder. Intrahepatic macrophages, also called Kupffer cells (KCs), are the key regulatory factors in the network of innate and adaptive immune interactions in the hepatic IRI mechanism. KCs are activated in early-stage IRI, resulting in the massive production of proinflammatory factors, cytokines, and ROS, which is a key link leading to hepatic microcirculation disorder (Tamura et al. 2012). Warm ischemia in DCD liver can cause acute liver injury and aseptic inflammation; macrophage activation induces increased endothelial ICAM-1 expression, which induces circulating neutrophil infiltration and accumulation, resulting in hepatic sinusoid stenosis with partial or complete occlusion, eventually causing hepatic microcirculation disorder. Furthermore, reperfusion after ischemia will aggravate liver damage (Konishi and Lentsch 2017; Quesnelle et al. 2015).

In the intermediary link that causes hepatic microcirculation disorder, the main factors associated with hepatic sinusoid microcirculation disorder are ICAM-1, VCAM-1, and vWF. ICAM-1 is a product of the inflammatory response, and is mainly expressed in SECs; it is the main sinusoidal neutrophil ligand and a neutrophil adhesion and infiltration marker, which indicates the degree of hepatic sinusoid congestion (Farhood et al. 1995). KC depletion can prevent DCD liver reperfusion injury, inhibiting ICAM-1 expression significantly, and ICAM-1 deletion reduces neutrophil recruitment to the liver (Frankenberg et al. 1998; Nishimura et al. 1996). VCAM-1 is mainly expressed in hepatic SECs and vascular endothelial cells, and is induced by ROS and proinflammatory cytokines such as tumor necrosis factor alpha (TNF-α) (Cook-Mills et al. 2011). We found that BP preservation inhibited ICAM-1 and VCAM-1 expression significantly superior to that of SCS and NMP, suggesting that BMMSCs inhibit neutrophil infiltration, endothelial cell activation, and the alleviation of hepatic sinusoid stasis and occlusion. This is consistent with the BMMSCs inhibiting DCD liver macrophage activation and improving the cascade of hepatic sinusoid stasis. Furthermore, ICAM-1 and VCAM-1 are characteristic molecules of endothelial dysfunction (Zonneveld et al. 2014), which also illustrates the superior effects of BMMSCs for reducing hepatic sinusoidal endothelial and vascular endothelial injury.

A polysaccharide protein found in plasma, subendothelial matrix, Weibel-Palade bodies of endothelial cells and platelet alpha granules, vWF bridges platelets, and the exposed collagen surface (Lenting et al. 2015). Increased vWF in the plasma of patients with acute liver failure and cirrhosis suggests poor prognosis (Reuken et al. 2015). When the endothelium is damaged, vWF binds to the subendothelial connective tissue, allowing the Weibel-Palade bodies to bind platelets with sufficient affinity (Sadler 1998). Therefore, the degree of intrahepatic vWF elevation suggests the degree of hepatic sinusoid congestion and endothelial damage. We found that BP preservation inhibited liver vWF expression significantly, with the lowest degree of sinusoid congestion and endothelial damage. During angiogenesis, BMMSCs interact with endothelial cells, which have excellent angiogenesis and endothelial repair properties, in an adjacent secretory and paracrine manner (Rahbarghazi et al. 2013; Fish and Hajjar 2015). The inhibitory effect of BMMSCs on vWF, ICAM-1, and VCAM-1 fully demonstrates the BMMSC protective effects on hepatic sinusoid microcirculation and endothelial injury.

TM is a transmembrane protein expressed on endothelial cell surfaces and plays an important role in regulating inflammation and intravascular coagulation (Ito et al. 2016; Ke 2017). In hepatic IRI, TM reduces neutrophil accumulation by inhibiting leukocyte expression of adhesion molecules (Fujii et al. 2018), and improves hepatic microcirculation. Detecting perfusate TM levels, we found that BMMSCs plus NMP significantly promoted TM expression, confirming that BMMSCs inhibit liver macrophage activation and improve endothelial damage. PAF is an important factor involved in hepatic IRI pathogenesis. ROS activates PAF during reperfusion, promotes neutrophil infiltration, and ultimately leads to IRI and microcirculation disorder. PAF inhibitors or its receptor inhibitors are effective for reducing macromolecular extravasation during ischemia or at the beginning of reperfusion, and for reducing IRI (Cicco et al. 2005; Serizawa et al. 1996; Noel et al. 1996). Here, BMMSCs inhibited PAF expression as compared with NMP alone. By detecting TM and PAF, which have important regulatory effects on neutrophil aggregation and infiltration, we verified the role of BMMSCs in inhibiting neutrophil aggregation and infiltration and improving hepatic microcirculation.

Finally, the vasoconstrictor ET-1 and the vasodilator NO are the main influencers of microcirculation perfusion. The balance between the two plays an important role in in maintaining hepatic microcirculation homeostasis. ET-1 is mainly produced by vascular endothelial cells and is the strongest endogenous vasoconstrictor currently known; ischemia and hypoxia are important stimuli for upregulating ET-1 (Davenport et al. 2016). When the liver is damaged, endothelial cell ET-1 expression is increased and NO production is decreased; the balance between the contraction and vasodilation of hepatic sinusoid and vessels is disrupted, and ET-1 contraction dominates, causing vasoconstriction, hepatic sinusoid stasis, increased intrahepatic vascular resistance, upregulated leukocyte–endothelial cell interaction, and portal hypertension (Feng et al. 2009; Rosado et al. 2012). NO relaxes the smooth muscles, dilating vessels, improving microcirculation perfusion, and inhibiting platelets, leukocyte adhesion, and antioxidation (Pacher et al. 2007). The NOS affecting liver microcirculation are mainly eNOS and iNOS. eNOS is continuously expressed only in liver endothelial cells, mainly maintaining physiological levels; iNOS is mainly induced by inflammatory stimulation, which once expressed, will synthesize a large amount of NO. Excessive increase of NO will promote peroxynitrite (ONOO^−^) production, resulting in endothelial dysfunction (Pacher et al. 2007). The present results show that BMMSCs promoted increased eNOS synthesis while inhibiting iNOS synthesis, and inhibited excessive NO production. Some studies have confirmed that eNOS-derived NO improves IRI, while iNOS-derived NO promotes it (Wang et al. 1998; Isobe et al. 1999). BMMSC regulation of NOS is closely related to NOS inhibition of macrophage activation, inhibition of macrophage synthesis of large amounts of iNOS and NO, and the improvement of endothelial injury. Therefore, we believe that BMMSCs plus NMP has a better effect for improving DCD liver microcirculation perfusion than NMP, which is even better than SCS.

It should be noted that the shortcoming of the present study is that the NMP system is a single circulation system without a drainage device. Excessive perfusion time limits the level of organ repair of the perfusion system. Next, we will improve the perfusion method for further research.

## Conclusion

In the situation of a shortage of liver donors, DCD liver is an effective method for expanding the available donor pool. BMMSCs combined with NMP can inhibit hepatic sinusoid congestion and endothelial injury by inhibiting intrahepatic macrophage activation and intercellular adhesion in rats, and regulate the ET-1/NO balance to improve DCD liver perfusion and microcirculation. The present study reveals the protective factors for improving DCD liver quality and provides experimental evidence for the use of clinical DCD liver. Here, we explored the improvement of DCD liver quality by BMMSCs plus NMP pre-transplantation; assessing recipients’ quality of life after transplantation would be more important. Therefore, our future research direction is whether the preservation system can improve transplant recipient quality of life or prolong survival time.

## Electronic supplementary material

ESM 1 (DOC 534 kb)
