# Supplementary material for: Bone marrow mesenchymal stem cells combine with normothermic machine perfusion to improve rat donor liver quality—the important role of hepatic microcirculation in donation after circulatory death
Source: Cell Tissue Res. 2020 Apr 29;381(2):239–54. doi: 10.1007/s00441-020-03202-z (PMC7369267; doi:10.1007/s00441-020-03202-z)
Supplement: Supplementary file 1 — (DOC 534 kb) [file 441_2020_3202_MOESM1_ESM.doc]

**Figure legend**

**
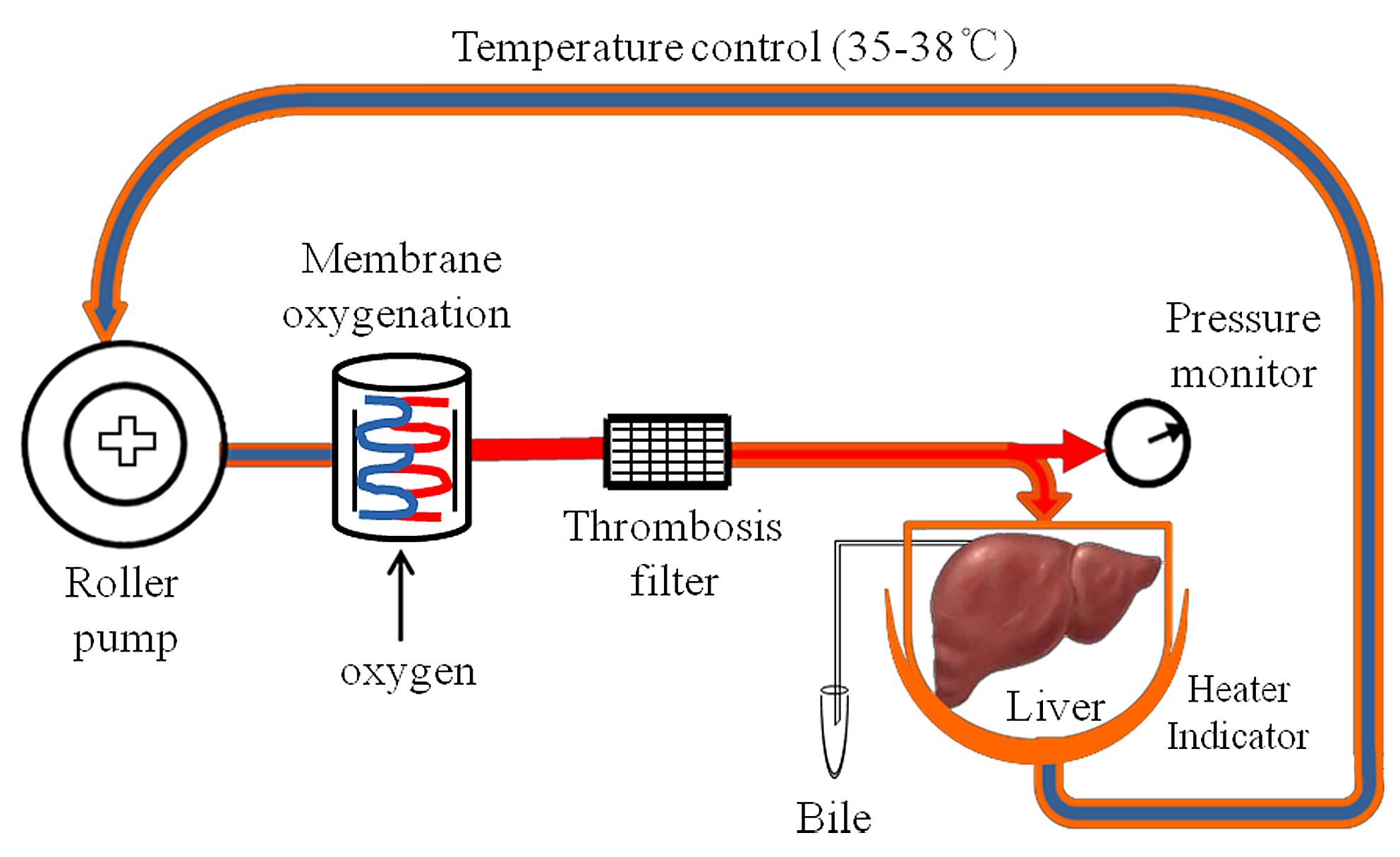
**

**Supplementary fig. 1** Schematic diagram of the NMP system for preserving DCD liver. Abbreviations: DCD, donation after circulatory death; NMP, normothermic machine perfusion
